# Global perspectives of the impact of the COVID-19 pandemic on learning science in higher education

**DOI:** 10.1371/journal.pone.0294821

**Published:** 2023-12-07

**Authors:** Shima Salehi, Cissy J. Ballen, Klara Bolander Laksov, Khayala Ismayilova, Philip Poronnik, Pauline M. Ross, Vicky Tzioumis, Carl Wieman

**Affiliations:** 1 Graduate School of Education, Stanford University, Stanford, CA, United States of America; 2 Department of Biological Sciences, Auburn University, Auburn, AL, United States of America; 3 Centre for the Advancement of University Teaching (CeUL), Stockholm University, Stockholm, Sweden; 4 KTH Royal Institute of Technology, Department of Learning in Engineering Sciences, Stockholm, Sweden; 5 School of Medical Science, Faculty of Medicine and Health, The University of Sydney, Sydney, New South Wales, Australia; 6 School of Life and Environmental Sciences, Faculty of Science, The University of Sydney, Sydney, New South Wales, Australia; 7 School of Physics, Faculty of Science, The University of Sydney, Sydney, New South Wales, Australia; 8 Department of Physics and Graduate School of Education, Stanford University, Stanford, CA, United States of America; Ahvaz Jundishapur University: Ahvaz Jondishapour University of Medical Sciences, ISLAMIC REPUBLIC OF IRAN

## Abstract

The COVID-19 pandemic required higher education institutions to rapidly transition to Emergency Remote Instruction (ERI) with little preparation. Discussions are now underway globally to learn the lessons of COVID-19 and to use this knowledge to shape the future of learning science in higher education. In this study, we examined the experiences of instructors and students to ERI in three universities across three continents–America, Europe, and Australia. We measured the instructional strategies used by instructors including assessment types, and interaction opportunities during and outside class schedules. We also measured the learning challenges experienced by students including planning, distractions, technology, learning resources, their views on educational quality and what characterized quality interactions during ERI. Our findings suggest that most instructional strategies used by instructors changed little during ERI, although the nature of instructor and student interactions during class relied more heavily on technology. Students reported significant learning challenges which included distractions from their physical and social media environments and access to technology. Both instructors and students reported that interactions with each other and their peers were concerningly low, albeit similar to pre COVID-19 pandemic levels. There were differences in the perceptions of instructors and students on whether instructor-student interactions were better or worse online. Common among all universities, there was a large proportion of students reporting mental health and work-related stress. Lessons to be learned from the COVID-19 pandemic include ensuring more support for instructors to implement effective and equitable pedagogies and an increased recognition of the importance of practicals, and the social, interactive and hands-on aspects of learning science in higher education. We predict that the incorporation of active learning pedagogies and strategies which increase student engagement and foster a sense of belonging will be ongoing global challenges for learning science in a post COVID-19 campus.

## Introduction

The COVID-19 pandemic created immense challenges, disruption and opportunities for the higher education sector [1, 2]. Almost overnight, universities across the globe ceased on campus teaching and assessment and moved to Emergency Remote Instruction (ERI) [1]. Along with these challenges also came opportunities for higher education to learn from the experiences of instructors and teaching assistants, students, and administrators during the COVID-19 pandemic and ERI [2]. In a post COVID-19 campus we need to identify and retain learning strategies which were effective and at the same time recognize the limitations of ERI, especially for practical disciplines such as Science. Key questions include: “What learning strategies should we leave behind and what should we carry forward into the future?” “How do we create more positive outcomes from a period of time when instructors and students have experienced great adversity with impacts on their mental health and well-being [1–5]”? To answer these questions effectively, we need to understand the teaching strategies used by instructors during ERI and the responses of students to these strategies. If we are to generalize broadly then we need a global perspective and understanding.

A wide range of studies have described the challenges and opportunities that the COVID-19 pandemic and ERI have posed for online teaching and learning [4–11] Prior to the COVID-19 pandemic, almost a decade ago, some commentators warned online education was a *tsunami* coming for higher education and that one should prepare. Although there was a dramatic increase in online learning and course offerings, the *tsunami* did not really arrive until the changes forced by the COVID-19 pandemic. Some studies suggest that the COVID-19 pandemic may have accelerated online learning and digital transformation more than any other event in higher education, including the boom and fascination around Massive Open Online Courses (MOOCs) a decade ago [2, 3, 9–12].

During the decade prior to the COVID-19 pandemic, a substantial body of research focused on the elements which influenced student satisfaction in online environments [13–15]. For example, Kuo et al. [13] found that “learner-instructor interaction, learner-content interaction, and Internet self-efficacy were significant predictors of student satisfaction in fully online learning settings, while learner-learner interaction and self-regulated learning did not predict student satisfaction” [13, p.33].

In addition to these aspects, Cole et al. [14] found “issues of timeliness and instructor’s accessibility were also raised but to a lesser extent than clarity and instructor’s ability to effectively use technology in online courses” [14, p. 123]. Furthermore, and similar to Kuo et al. [13], Cole et al. [14] and Bolliger and Martindale [16] identified three key factors central to online student satisfaction: 1) instructor quality, 2) technology, and 3) interactivity. They stated that instructor quality and skills in “communication, feedback, preparation, content knowledge, teaching methods, encouragement, accessibility, and professionalism” were key to student satisfaction [16, p. 65]. Moreover, students needed reliable technology that could be used “early and often” and opportunities for interaction so that they felt “involved and stay engaged” [16, p.65]. Similarly, Castro and Tumibay [17], in a meta-analysis of online learning, found that instructional design and the active role of institutions through support of staff and students was critical in increasing efficacy. They noted that “important course design characteristics that shape learning experiences are flexibility, personalization, varied forms of assessment, small group learning with designed interactions, and technologies and media which used an adopted mix of pedagogies” [17, p. 1383].

During the COVID-19 pandemic, Rapanta et al. [5] reported that the most effective learning designs combined the cognitive, social, and interactive environments and adapted assessment. In detail, the cognitive environment should include a context (i.e., the learners’ goals), the social environment should include communication channels and concrete tasks, and the interactive environment should encourage participation and peer collaborations (5, p. 937). Several researchers conclude that central to successful online learning is the design of learning environments where students interact with content, as well as with each other and instructors or faculty [2, 16, 17].

ERI is dramatically different from carefully designed online education with purposely selected tools that aid educational objectives [1, 2, 18]. Rapanta et al. [5] also found that the main challenge of ERI was “the urgent and unexpected request for previously face-to-face university courses to be taught online” [5, p. 923]. Many instructors were unprepared for ERI and in many cases were not offered support or training to aid their efforts [19, 20]. Where instructors were provided with support and training in course design, communication, time management and technology, however, outcomes were significantly improved [21].

The many lessons learned from the sudden pivot to ERI can inform us in a post COVID-19 context to better design future online and on-campus education environments. In fact, Rapanta et al. [5] note that the “worst thing that could happen is not learning from the crisis we experienced” and that we need to respond to the COVID-19 pandemic taking away with us the “lessons learnt” (5, p. 941).

For instructors, lessons learned from ERI in response to the COVID-19 pandemic include the benefits from integration of digital pedagogies to remediate inequalities around access to learning material, the value of continued novel pedagogical experimentation and innovative digital assessment, and the need for on-going reflexive practice [3, 4, 8, 22]. In addition, they included challenges (for most instructors) which caused mental health and work-related stress, digital fatigue, and negative impacts on work-life balance [3, 4, 23] and which required resilience [24].

The COVID-19 pandemic and ERI were also exceptionally disruptive and stressful for students. The pandemic upended students’ daily routines and subjected them to unusual isolation [18]. In many situations, online education infrastructure and student access to good remote learning settings was suboptimal for teaching and learning due to inequitable access to technology and the internet [25–27]. This was particularly notable in disciplines such as Science where laboratories and peer to peer discussions and interactions are important in learning.

## This study

The aim of this study was to gain an understanding of the instructional strategies used by instructors, the experiences of students, and the interactions between instructors and students across three higher education institutions from three continents–America, Europe, and Australia–that shaped educational quality in the wake of the global disruption of the COVID-19 pandemic.

This was done to gain global understandings and insights of instructor and student experiences of effective and ineffective teaching strategies, the frequency of instructor to student and student to student interactions, and the overall experiences that shaped learning environments in the wake of a global disruption. We used models of effective learning design provided by Rapanta et al. [5] and Bolliger and Martindale [16] which identified instructors, context and content, technology, and interactivity as central to online student satisfaction to explore two key research questions:

1. What learning designs did instructors use in ERI? That is, what were the instructional components, assessment types, and interaction opportunities used during ERI?
2. What were students’ experiences of ERI? That is, what were the challenges, which learning resources were effective, and what characterised quality interactions during ERI?

Although previous studies have reported on various teaching strategies for online learning used during the COVID-19 pandemic, we know less about how instructors and students responded to these strategies and pedagogies. A key purpose of this study was to understand these responses so that we may be able to use the COVID-19 pandemic and ERI as an opportunity for instructors to create more effective and equitable pedagogies and increase student engagement for learning science in higher education.

## Materials and methods

### Design of the study

#### Participating higher education institutions

In this study, instructors and students were surveyed about their experiences of ERI in three institutions on three continents: Stockholm University in Stockholm, Sweden, Auburn University in Alabama, United States, and the University of Sydney in Sydney, Australia. The characteristics of these institutions are summarized in Table 1. Each institution varied in the timeline of actions and responses to the COVID-19 pandemic, that is, when classes stopped, institutions closed and ERI commenced. There were similarities and differences in size and structure, semester length and student characteristics among the institutions which participated (Tables 1 and S1). Higher education institutions can also be considered in terms of their cultural constructs [28, 29]. In this study it was important to recognize the influence of local cultures on shared norms, values, and practices which develop between individuals, and which vary subtly among institutions in higher education and may lead to differences in instructor and student responses [30–32].

**Table 1:** Comparison between the size and structure, faculty system and standards, semester length, teaching resources and student catchment and characteristics at Auburn University, United States, Stockholm University, Sweden and The University of Sydney, Australia.

	Stockholm University, Sweden	Auburn University, Alabama, USA	The University of Sydney, Australia
Size and structure	• Sweden’s largest research university • Identifies as campus university in Swedish capital. • Publicly funded. • 27000 FTE students; • 5000 faculty. • Faculty of Science has five sections • 3800 students • 360 faculty • 600 grad studs	• Flagship university in Alabama. • Located in small college town of Auburn. • Publicly funded • 24600 FTE students; • 1330 faculty. • College of Sciences and Mathematics with five departments. • 2700 students • 200 faculty • 400 grad studs	• Australia’s oldest university. • Located in central Sydney, capital city in New South Wales, Australia. • -Publicly funded. • 73000 FTE students; 8100 faculty. Faculty of Science with eight schools and Faculty of Medicine and Health with a School of Medical Science. • 9564 students • 332 faculty • 790 grad studs
Faculty system and standards	Scientific and teaching portfolio when applying for position. No clear criteria Local requirement of 10 weeks HE training	Tenure track system. Move in rank based on student evaluations and faculty peer reviews.	Tenure track either in research, teaching and research, or education. Teaching standards framework are used to achieve fellowships
Semester Length	Two 20 week-semesters	Two 15-week semesters	Two 18-week semesters
Teaching resources	CeUL center of Ed development Courses + WS SoTL support Arenas of exchange	Biggio center Ed dev Instructional technology Learning exp design	Central unit responsible for educational innovation
Student catchment, location and characteristics	-Students live in own flats, student housing, or with family -Students are, on average, older than US and Australia institutions	-Students live on or near campus -Students move to Auburn from across the US (only 5% local)	-Most students are local and live with family (88%)
Modality of teaching before pandemic	Mostly in-person courses with lecture and to active learning in courses	Mostly in-person courses lecture and active learning used in courses	Mostly in-person courses lecture and active learning used in courses
Institutional Support and guidance for transitioning to ERI	Each department chose how to organize the teaching (no requirement). Zoom use supported by CeUL in courses, ‘cafés’ and drop-in sessions	Biggio center offered support for instructors transitioning to zoom to teach synchronously or asynchronously.	Each instructor chose how to organize the teaching synchronously. Zoom was supported by the university. There were central efforts to organize summative exams.

#### Survey

Across the three institutions, we collected survey data from students and instructors in science disciplines. To answer the first research question about the learning designs used by instructors in the pivot to ERI, an instructor survey was adapted from the *Teaching Practices Inventory* (TPI) which had been developed and validated by Wieman & Gilbert [33]. The instructor survey asked questions about the:

- general characteristics of the course such as size, number of credit hours, and characteristics of the student population (e.g., undergraduate or postgraduate level),
- instructional components of the course (e.g., types of assignments, synchronous or asynchronous lectures, recorded or unrecorded lectures),
- types and weights of different assessment types (e.g., weekly formative quizzes, summative exams),
- format and extent of student and instructor interaction opportunities,
- comfort and challenges associated with ERI.

To answer the second research question about the students’ experience of ERI, the student survey was designed to capture students’ responses about:

- educational experiences during ERI compared to face-to-face on campus instruction,
- learning environments during ERI, including distractions of various types, that impacted their educational experience,
- the type of coursework and amount of time students spent during scheduled class sessions and outside of scheduled class sessions (e.g., live lecture, workshop or tutorial class sessions, working on assessments and Teaching Assistant (TA) sessions), and
- type and extent of student interaction with their peers and instructional team during scheduled class sessions, and the factors influencing these interactions.

Given all the pressures and demands of the COVID-19 pandemic, both instructor and student surveys were designed to be minimally demanding and to be completed in less than 10 minutes. The student survey was tested through validation interviews with eight members of staff and eight undergraduate students at Stockholm University in Sweden and 20 undergraduate students in the United States. At Stockholm University, the surveys were translated into Swedish (and the responses were translated back to English for the purposes of dissemination in this research paper). In this process, the survey was tailored and adapted to conditions at Stockholm University, where the response to the COVID-19 pandemic differed in some ways to that of the United States and Australia. Hence, some questions were omitted or changed in the Swedish version of the survey. Table 2 lists the questions of each survey that were used and is aligned with the description of the results.

**Table 2:** List of questions asked in survey.

	Auburn University and The University of Sydney	Stockholm University
**A. Common Instructional Components**	1. Course activities students are expected to complete (Check all that apply) • Assignments (problem sets, projects, or writing assignments) with feedback from instructor, teaching assistant, or peers, with opportunity to redo work to improve grade • Assignments (problem sets, projects, or writing assignments) that are graded Graded exam(s) or quizzes during the term • Final exam • Reading assignments • Watching pre-recorded videos • Attending live virtual class with instructor and other students present • Attending live virtual class sessions in which students complete and turn in work Optional watching of recorded live virtual class with instructor • Self-evaluation of learning exercises • Other 2. Fraction pre-recorded videos with embedded questions for which student is marked for correctness • 0 • less than 10% • 10% to 30% • 30% to 50% • 50% to 70% • 70% to 100% 3. Fraction pre-recorded videos with embedded questions for which student is marked for completion • 0 • less than 10% • 10% to 30% • 30% to 50% • 50% to 70% • 70% to 100%	1. Which of the following course activities did you expect the students to complete during the course (select all relevant alternatives)? • Tasks (problem solving, projects or writing assignments) with (formative) feedback from teachers or other students. • Assessed and / or compulsory assignments that count towards the final grade of the course. • Reading assignments to be discussed at seminars • Listening to online lectures in real time • Watching videos recorded by teachers in advance • Watching video material recorded by external actors, such as other universities, publishers, companies, Khan Academy etc. • Having the opportunity to go back and voluntarily watch recorded seminars or lectures after they were conducted • Attend scheduled virtual seminars with teachers and other students present (eg in Zoom) • Participate in virtual teaching where students complete and submit (and possibly comment on each other’s) work, eg in Athena • Working in groups with course material • Making presentations for other course participants as a basis for feedback and / or discussion • Other 2. If the students were watching videos, did they have subsequent content questions where the students got feedback on their answers? • Always • Most of the time • Half of the time • Sometimes • Never • Not relevant—they did not watch videos
**B. Assessment**	1. Compared to when you taught the course in-person, not including the effect of the final exam, this weighting is • Similar • Different • Very Different 2. Different elements used to evaluate the students • Midterm Quizzes or exams • Final Exam • Homework assignments Paper(s) or project(s) In-class activities Participation • Other	1. Compared to when you taught the course on campus, the exam was: • Similar • Partly different • Very different • I did not teach this course before 2. The following activities were planned and used on the course to assess the students’ learning (Mark all relevant) • Dugga / quiz or half- course exam • Participation • Compulsory participation • Home exam • Essay or Project work • Monitored exam (salstenta) • Oral assessment • Other
**C. Interaction During Live Sessions**	1. Number of hours per week you do live online teaching, where all students are expected to be logged in and participating at the same time, via Zoom or similar technology • 0 • Less than 1 • 1 to 2 hours • 2 to 4 hours • 4 to 6 hours • More than 6 hours 2. Fraction of class time students are in small group discussions or problem-solving in Zoom breakout rooms (or similar technology) • 0 • Less than 10% • 10% to 30% • 30% to 50% • 50% to 70% • More than 70% 3. TAs supported online class sessions by monitoring and responding to student questions posted in chat box or other question asking formats. • Always • Most of the time • About half the time Sometimes • Never 4. Fraction pre-recorded by you (rather than from outside source (MIT Open Courses, Kahn Academy, etc.)) • 0 • less than 10% • 10% to 30% • 30% to 50% • 50% to 70% • 70% to 100% 5. Number of times in a typical class you poll students to check for understanding (using Zoom poll, poll everywhere, other) • None • 1 to 3 times • 3 to 5 times • 5 to 8 times • More than 8 times	
**D. Interaction Outside Class**	1. Opportunities for student-instructor interaction and feedback (Check all that apply) • Virtual drop-in office hours by instructor (Zoom or similar) • Virtual office hours by teaching assistants • Piazza or other discussion boards to which teaching team (you and/or TAs) regularly contribute Teaching team provided feedback on assignments and exams/quizzes • Instructor arranged one-on-one meetings with students 2. Fraction of students in class typically participating each week • 0 • Less than 10% • 10% to 30% • 30% to 50% • 50% to 70% • 70% to 100% 3. Fraction participating when course was taught in-person • 0 • Less than 10% • 10% to 30% • 30% to 50% • 50% to 70% • 70% to 100%	1. During the course, there were opportunities for student- teacher interaction where students could ask specific questions and get answers (select all relevant options) • In the beginning of / end of / during lectures and seminars • Through virtual drop-in times with the teacher (zoom or similar) • Via a message board or other discussion forum that teachers (you and / or other teachers) regularly read and responded to • Through teacher feedback on assignments, exams / quizzes etc • At meetings organized by me to meet students one by one
**E. Overall Instructor Experience**	1. I am now comfortable teaching online. • Extremely comfortable • Somewhat comfortable • Neither comfortable nor uncomfortable Somewhat uncomfortable • Extremely uncomfortable 2. I would prefer to not have to teach this course online in the future • Strongly agree • Somewhat agree • Neither agree nor disagree • Somewhat disagree • Strongly disagree	1. I am comfortable teaching this course online now • Extremely comfortable • Somewhat comfortable • Neither comfortable nor uncomfortable Somewhat uncomfortable • Extremely uncomfortable 2. I would prefer not to have to teach this course online in the future • Strongly agree • Somewhat agree • Neither agree nor disagree • Somewhat disagree • Strongly disagree
**F. Planning**	1. I have a well-planned daily schedule for my coursework • Strongly agree • Somewhat agree • Neither agree nor disagree • Somewhat disagree • Strongly disagree 2. I was able to stick to my planned schedule. • Strongly agree • Somewhat agree • Neither agree nor disagree • Somewhat disagree • Strongly disagree 3. It was easier to stick to a daily schedule with courses that have scheduled live class sessions than when I was watching recorded videos or class sessions. • Strongly agree • Somewhat agree • Neither agree nor disagree • Somewhat disagree • Strongly disagree	1. I planned my distance studies using a daily structure that I created myself • Strongly agree • Somewhat agree • Neither agree nor disagree • Somewhat disagree • Strongly disagree 2. I was able to follow my study planning • Strongly agree • Somewhat agree • Neither agree nor disagree • Somewhat disagree • Strongly disagree 3. It was easier to follow my study planning when teaching was on campus than when it was conducted online • Strongly agree • Somewhat agree • Neither agree nor disagree • Somewhat disagree • Strongly disagree
**G. Distractions**	1. The following reduced my ability to participate/perform in the course: • Limited internet access • Distractions from physical environment where I was online from • Distractions from my personal technology (phone, computer, etc.) • Time zone differences • Health or work-related stress (yours or family/roommate) • Other	1. The following factors hampered my participation in the online course (mark all relevant) • Limited internet access • Problems with my technical equipment (telephone, computer etc.) • Distracting factors in my physical study environment • My health • Health- or study-related stress • Related person(s) situation • Lack of good agronomical conditions (physical working environment conditions) • Lack of social contact with students and teachers • Other factors
**H. Technology**	1. I am familiar and comfortable with all the technology needed for my online courses. • Strongly agree • Somewhat agree • Neither agree nor disagree • Somewhat disagree • Strongly disagree	1. I felt comfortable using the technology needed to follow the course online • Strongly agree • Somewhat agree • Neither agree nor disagree • Somewhat disagree • Strongly disagree
**I. Education Quality**	1. My overall educational experience in this course compared to the average of my previous in- person college courses in similar disciplines was Not asked at The University of Sydney • Much better • Somewhat better • About the same • Somewhat worse • Much worse	1. My overall experience of the course compared to campus- based courses was that it was • Much better • Somewhat better • About the same • Somewhat worse • Much worse
**J. Learning Resources**	1. Rank the top three in terms of their contribution to your learning, with 1 being greatest contribution: (Drag line of text to reorder ranking) • Attending live online classes • Watching recorded course videos • Doing course assignments (homework, writing, etc.) • Reading of textbook or assigned texts • Watching or reading outside reference materials (Khan Academy, Wikipedia, MIT open courses …) • Study groups • Office hours • Other	1. The following activities contributed greatly to my learning and commitment • Attendance of scheduled online teaching with teachers and other students present (eg in zoom). • Watched pre-recorded videos. • Answered questions from teachers about the content of lectures. • Read course literature • Worked with less in-depth assignments (eg problem solving, smaller writing assignments) • Worked on major individual project or essays • Worked on group project • Full-class discussion • Other study-related activities planned by the teacher • View or read other reference material (Khan Academy, Wikipedia, open source material, MOOCs, etc.) • Self studies • Informal work in group (not organized by the teachers) • Informal meeting with teachers e.g. at ’drop-in’ time about my study situation • Other
**K. Instruction Quality**	1. The instructor had a good sense of my level of understanding of the course material. • Strongly agree • Somewhat agree • Neither agree nor disagree • Somewhat disagree • Strongly disagree 2. I felt that the feedback I received in this course from the instructor and TA’s (including on assignments, during class, in virtual office hours, from exams or quizzes, etc.) helped me to succeed. • Strongly agree • Somewhat agree • Neither agree nor disagree • Somewhat disagree • Strongly disagree	1. The teacher had a good idea of how well I understood the course content and how well I kept up with the course. • Strongly agree • Somewhat agree • Neither agree nor disagree • Somewhat disagree • Strongly disagree 2. I felt that the feedback I received from a teacher or other students during the course helped me to succeed in the course. • Strongly agree • Somewhat agree • Neither agree nor disagree • Somewhat disagree • Strongly disagree
**L. Interactions**	1. I felt comfortable asking questions in live online classes. • Not applicable • Strongly agree • Somewhat agree • Neither agree nor disagree • Somewhat disagree • Strongly disagree 2. My comfort level in asking questions in this online unit compared to my previous on-campus classes was: • Much better • Somewhat better • About the same • Somewhat worse • Much worse 3. I regularly or occasionally asked questions (choose all that apply). • During breakout sessions • In whole class discussions via Zoom or similar • In whole class discussions via raising hand • I did not ask questions at all 4. I interacted with the lecturer outside live online class time through (choose all that apply). • There were no live online classes in this unit • I did not interact with my lecturer • Attending their virtual office hours • Zoom meeting with them outside office hours • Communicated regularly via discussion board, Slack etc. • Email • Other 5. I interacted with the tutors outside live online class time through (choose all that apply). • I did not interact with my tutors • Attending their virtual office hours • Zoom meeting with them outside office hours • Communicated regularly via discussion board, Slack, email, etc. • The unit did not have a tutor 6. Percentage of unit related study or work time outside of class spent with other students in the unit. • Less than 10% • 10% to 30% • 31% to 50% • 51% to 70% • More than 70% 7. Ease of my interactions with the instructor in this online course compared to my previous in-person college courses in similar disciplines was • Much better • Somewhat better • About the same • Somewhat worse • Much worse 8. Ease of interacting with other students in this online course compared to my previous in-person college courses in similar disciplines was • Much better • Somewhat better • About the same • Somewhat worse • Much worse	1. I felt intellectually engaged when the course was taught online. • Strongly agree • Somewhat agree • Neither agree nor disagree • Somewhat disagree • Strongly disagree 2. I participated actively by, for example, asking questions, asking to speak, contributing to group discussions • Strongly agree • Somewhat agree • Neither agree nor disagree • Somewhat disagree • Strongly disagree 3. I interacted with other students on the course outside the teaching / assignments organized by the teacher. (select if applicable) 4. Interaction on Athena or other learning platform with the teacher • Was much lower • Was somewhat lower • Was about the same • Was somewhat higher • Was much higher • Not applicable 5. Interaction on Athena or other learning platform with other students • Was much lower • Was somewhat lower • Was about the same • Was somewhat higher • Was much higher • Not applicable

#### Survey distribution

The surveys were distributed online. Most of the students who were surveyed were enrolled in undergraduate courses (Auburn University 89%, Stockholm University 53%, the University of Sydney 93%), and most were at first- and second-year level (Auburn University 57%, Stockholm University 47%, the University of Sydney 73%). The total number of students who were surveyed and answered all the questions was 1,856 (960, 775, and 121 at Auburn University, Stockholm University and The University of Sydney respectively). Students at postgraduate level were also surveyed at Stockholm University. Surveys were done by students in small class sizes at Stockholm and Auburn Universities. At these universities, 69% and 61% of the courses had enrollments of fewer than 50 students. At The University of Sydney most instructors taught larger classes, and only 18% taught classes with fewer than 50 students. While Stockholm University and The University of Sydney collected data on student gender, Auburn University did not. The total number of instructors who were surveyed and answered all the questions was 293 (83, 137, 73 at Auburn University, Stockholm University and The University of Sydney respectively) (Table 3).

**Table 3:** Total number and gender of students and instructors surveyed at Auburn University, United States, Stockholm University, Sweden and The University of Sydney, Australia. N/A indicates the option was not included in the survey.

	Stockholm, Sweden		Auburn, USA		Sydney, Australia	
	Students	Instructors	Students	Instructors	Students	Instructors
Female	436	50	N/A	N/A	61	17
Male	317	84	N/A	N/A	24	27
Did not identify with either gender/ Refrained from answering	22	3	N/A	N/A	36	29
Total	775	137	960	83	121	73

#### Data analysis

Responses from instructors and students were first cleaned and organized as excel files for each institution separately. Then, we used the CSV format of the data to examine descriptive statistics and/or statistical analysis in the R studio. T-tests were used to determine any statistically significant differences between binary variables such as gender, and prior versus during ERI. If in any case, the data for statistical analysis diverged significantly from the normal distribution, we also verified the statistical analysis by conducting non-parametric Wilcoxon tests

#### Ethics

At each institution a lead academic was responsible for the ethical and regulatory requirements related to the protection of human participants in research. Ethics approval for this study was provided by the Office of Human Research at Auburn University project number IRB#20–189 EX 2004. At The University of Sydney ethics approval was provided by the Human Research Ethics Committees (HRECs) project number 2020/491. At Stockholm University ethics approval was provided by the Stockholm Regional Ethics Committee, project number 2020–04348. A consent form including information about the purpose of the project preceded the online survey and responses were anonymous.

## Results

### Responses of instructors

Instructor responses were collected to answer Research Question 1: What learning designs did instructors use in ERI? That is, what were the instructional components, assessment types, and interaction opportunities used during ERI? As detailed in Table 2, the instructor survey included items about aspects of ERI including the instructional components, assessment types and weightings, and opportunities for student interaction with instructor and peers. Opportunities for ‘interaction’ included those that occurred during and outside of classes.

The instructional components most commonly and consistently used across all universities were graded assignments with feedback and live sessions (Questions: Table 2-row A-Q1, Results: Table 4). Although videos and recorded live sessions were made available fewer than half of the instructors expected students to watch pre-recorded videos (Table 4). Live sessions did not generally include submissions of work. Assignments with formative feedback were commonly used by Stockholm University (82%). A small proportion of instructors at Auburn (13%) and Sydney (10%) who pre-recorded their videos included embedded questions with feedback on correctness in contrast to 40% who did so at Stockholm (Questions: Table 2-row A-Q2&3).

**Table 4:** Instructional components used by faculty during ERI at Auburn University, United States, Stockholm University, Sweden and The University of Sydney, Australia. Data is percentage and there was a total number of 293 responses.

Instructional components	Stockholm (n = 137)	Auburn (n = 83)	Sydney (n = 73)
Assignments with formative feedback	82%	31%	32%
Graded assignments	69%	57%	70%
Readings	37%	35%	16%
Watching pre-recorded videos	40%	39%	40%
Live sessions	73%	49%	68%
Live sessions with students turning in work	37%	13%	21%
Recording of live sessions	42%	28%	45%

Generally, instructors did not make changes to the assessment type or weighting during ERI. Assessment modes included a mixture of midterm quizzes or exams, final exams and homework assignments and projects (Questions: Table 2-row B-Q1, Table 5). Students at Stockholm University were often assessed at the end of each 5-week course, while assessment types at Auburn University and The University of Sydney included a mixture of midterm quizzes or exams, final exams and homework assignments and projects. For Auburn University, the two most common assessment types were midterm quizzes and exams, and final exams. At Stockholm University the most common assessment types were homework assignments, papers and projects, and in-class activities. At The University of Sydney, the most common assessment type was final exams, while midterm quizzes and homework assignments were equally common. Instructors did not significantly change the weighting of assessments (Questions: Table 2-row B-Q2, Results: Table 5).

**Table 5:** Assessment type and weighting used by faculty during ERI at Auburn University, United States, Stockholm University, Sweden and The University of Sydney, Australia. Data is percentage and there was a total number of 293 responses. *Monitored exam.

Assessment	Stockholm (n = 137)	Auburn (n = 83)	Sydney (n = 73)
Midterm quizzes or exams	23%	94%	40%
Final exam	17% *	89%	55%
Homework assignments	61%	76%	42%
Papers or projects	56%	43%	34%
In class activities	61%	30%	27%
Participation	48%	29%	19%
Assessment weighting same or very similar	85%	93%	93%

There were synchronous interactions between academics and students during ERI. In the surveys distributed at Auburn University and The University of Sydney instructors were asked about opportunities for synchronous interactions between instructors and students during live sessions in ERI. At Auburn University, ∼50% of instructors delivered 1 to 4 hours of live synchronous teaching sessions per week, while 30% of instructors did not deliver any. In contrast, 63% of instructors at The University of Sydney delivered 1 to 4 hours of synchronous live sessions per week (Questions: Table 2-row C-Q1). During the delivery of live sessions, the use of breakout room discussions and group work also varied across universities. At Auburn University, 70% of instructors never used breakout rooms. In contrast, instructors at The University of Sydney frequently used breakout room discussions, with only 21% of instructors reporting that they never used them (Questions: Table 2-row C-Q2).

There were also differences between Auburn University and The University of Sydney in monitoring and responding to the chat function in Zoom which formed a significant portion of synchronous live sessions. At Auburn University, 57% of the instructors reported that they never monitored the chat. In contrast just 17% of instructors at The University of Sydney reported that they never monitored the chat while 57% of instructors reported that they always monitored the chat option (Questions: Table 2-row C-Q3). Instructors at both institutions who reported that they delivered synchronous live sessions also indicated that they delivered pre-prepared lectures asynchronously, some for more than 50% of the classes (53% at Auburn University and 39% at The University of Sydney) (Questions: Table 2-row C-Q4). When instructors were asked whether they occasionally checked students understanding in these pre-prepared lectures, 47% of instructors at Auburn University and 48% of instructors at The University of Sydney reported that they never checked student understanding of the pre-prepared lectures (Questions: Table 2-row C-Q5).

At all three universities questions were asked about the types of interactions between instructors and students outside of class, including feedback on assignments or exams and virtual office hours (Questions: Table 2-row D-Q1, Results: Table 6). At Stockholm University and The University of Sydney, instructors’ interactions with students occurred through feedback on assignments and/or exams. At all three universities, instructors or teaching assistants (TAs) also had interactions with students during virtual office hours (Questions: Table 2-row D-Q1, Results: Table 6). The discussion board was not a common method of interaction at Auburn University, while for Stockholm University it was the second most common method and for The University of Sydney it was the third most common (Questions: Table 2-row D-Q1, Results: Table 6).

**Table 6:** Interactions between instructors and students used by faculty outside of class sessions during ERI at Auburn University, United States, Stockholm University, Sweden and The University of Sydney, Australia. Data is percentage and there was a total number of 293 responses. N/A indicates the option was not included in the survey.

Interactions between instructors and students	Stockholm (n = 137)	Auburn (n = 83)	Sydney (n = 73)
Instructor’s virtual office hour	57%	55%	45%
Teaching Assistant virtual office	N/A	30%	22%
Discussion boards	74%	7%	42%
Feedback provided on assignment and/or exams	87%	23%	51%
Instructors one-on-one meeting with students	39%	43%	22%

The level of interactions between instructors and students and attendance to office hours prior to and during ERI was similar and low. At Auburn University, 57% of instructors reported that fewer than 10% of students attended weekly office hours (prior to ERI) which was similar to 54% of instructors who reported that fewer than 10% of students attended virtual office hours during ERI (p = 0.70). At the University of Sydney, 53% of instructors reported that fewer than 10% of students attended weekly office hours (prior to ERI) compared to 43% who reported that fewer than 10% of students attended virtual office hours during ERI (p = 0.20) (Questions: Table 2-row D-Q2&3). Given that instructor office hours are one of the most common interaction opportunities instructors provided outside class sessions, it was alarming that fewer than 10% of students used them, particularly during ERI, when live interactions were not possible.

Finally, instructors at all three institutions reported that they were comfortable teaching online (72% Auburn University, 82% at Stockholm University, and 69% at The University of Sydney). Despite the reported comfort, the majority reported they preferred not to teach online, with 53%, 62% and 55% of instructors agreeing or strongly agreeing with this sentiment (Questions: Table 2-row E-Q1&2).

### Responses of students

Student responses were collected to answer research question 2: What were students’ experiences of ERI? That is, what were the challenges, which learning resources were effective, and what characterized quality interactions during ERI?

#### Planning

At all three universities, most students indicated that they had and followed a daily plan during ERI. While there was no difference in student planning related to gender at The University of Sydney (p = 0.75), we did observe gender differences at Stockholm University. Here, female students reported creating a plan more often compared to male students (p< 0.001), but we did not observe a gender difference in following a plan (p = 0.54). Overall, students at Stockholm University more commonly had and followed a daily learning plan compared to students at Auburn University and The University of Sydney (Questions: Table 2-row F-Q1&2, Results: Table 7). The majority of students at Auburn University and The University of Sydney also indicated that during the COVID-19 pandemic it was easier to plan for learning when they could attend in-person and live synchronized classes (Table 7). More than half of the students found that it was easier to follow the plan when the courses were synchronous (Questions: Table 2-row F-Q3, Results: Table 7). Students at Stockholm University reported it easier (49%) to follow their plan for in-person courses (Table 7).

**Table 7:** Student planning for learning during the COVID-19 pandemic at Auburn University, United States, Stockholm University and The University of Sydney, Australia. Data is percentage and there was a total number of 1,856 responses. N/A indicates the option was not included in the survey.

Student planning	Stockholm (n = 775)	Auburn (n = 960)	Sydney (n = 121)
Had a daily plan	73%	56%	52%
Could follow daily plan	75%	40%	42%
Easier to follow plan live in-person class	49%	N/A	N/A
Easier to follow plan with synchronized courses	N/A	59%	57%

#### Distractions

The most frequently cited sources of distractions for student learning were the physical environment followed by social media and mental health and work-related stress (Questions: Table 2-row G-Q1, Results: Table 8). This was lower for students at Stockholm University (32%) compared to students at Auburn University (66%) and The University of Sydney (62%) (Table 8). This may reflect the more relaxed COVID-19 regulations in Sweden. Students in Stockholm were not required to shelter in place or had hard ‘lock downs’ for extended periods. Instead, students at Stockholm University retained access to several locations that were shut down for students at Auburn University and The University of Sydney (e.g., cafes, libraries). Issues with internet access were also less distracting for students at Stockholm University (11%) compared to students from Auburn University (41%) and The University of Sydney (36%) (Questions: Table 2-row G-Q1, Results: Table 8). Differences in time zones was the least cited distractor for students at Auburn University and The University of Sydney (27% and 12% respectively). As students at Stockholm University are mainly in Sweden, which has one time zone, this question was omitted from the Stockholm survey.

**Table 8:** Sources of distractions for student learning during the COVID-19 pandemic at Auburn University, United States, Stockholm University and The University of Sydney, Australia. Data is percentage and there was a total number of 1,856 responses. N/A indicates the option was not included in the survey.

Sources of distractions	Stockholm (n = 775)	Auburn (n = 960)	Sydney (n = 121)
Physical environment	51%	81%	69%
Social media	N/A	71%	69%
Mental health and work-related stress	32%	66%	62%
Internet access	11%	41%	36%
Time zone	N/A	27%	12%

#### Technology

Overall, technology was not a major barrier for learning science for many students. High percentages of students across the three institutions reported that they did not have major technical difficulties and were comfortable using the technological platforms on which their online courses were offered (Questions: Table 2-row H-Q1, Results: Stockholm University = 84%, Auburn University = 76%, The University of Sydney = 77%).

#### Education quality

Many students reported that their education quality was reduced during ERI. When students were asked to compare their overall online education experience with the in-person experience (Questions: Table 2-row I-Q1), 47% of students at Stockholm University reported it was worse and 27% reported it was similar. There was no gender difference. Similarly, at Auburn University, 55% of students reported it was worse and 31% reported it was similar.

#### Learning resources

Students at The University of Sydney reported that the three most effective learning resources were videos (72%), assignments (71%) and live online lectures (72%) (Questions: Table 2-row J-Q1, Results: Table 9). The least effective learning resources were office hours followed by study groups (11 and 6% respectively). This result was not surprising given students did not have access to well-structured study groups during ERI.

**Table 9:** Rankings of the most and least effective learning resources which contributed to student learning during the COVID-19 pandemic at Auburn University, United States, Stockholm University and The University of Sydney, Australia. Data is percentage and there was a total number of 1,856 responses.

Ranking	Stockholm (n = 775)	Auburn (n = 960)	Sydney (n = 121)
**Most effective**			
Video	43%	80%	72%
Assignments	N/A	71%	71%
Live Online Lectures	56%	56%	72%
Reading	57%	0% Not among the most effective	0% Not among the most effective
**Least effective**			
Study Groups	20%	12%	6%
Office Hours	6%	6%	11%

Students at Stockholm University reported reading as one of the most effective learning resources, this contrasted with Auburn and The University of Sydney, where reading was not among the top three selected effective resources. At Stockholm and Auburn universities, 56% of students indicated that live online lectures were effective for their learning, while at the University of Sydney this was 72%. The least effective learning resources at all three institutions were office hours and study groups. Given the literature on the value of study groups and students’ reported desire for interaction with peers, this result may have emerged because students did not have access to well-structured study groups during ERI.

#### Instruction quality

Instruction quality was important for student learning. Students expressed dissatisfaction with the instructor’s knowledge about student understanding of the course material (Questions: Table 2-row K-Q1). At Stockholm University 41% of students indicated that their instructor did not have a sense of their understanding compared to Auburn University and The University of Sydney where 35% and 29% of students indicated that their instructor did not have a sense of student understanding of the course material. Also, a large portion of students did not find instructors’ feedback helpful for their learning (Questions: Table 2-row K-Q2). At Stockholm University, which relied on providing student feedback as a main source of instruction (Table 4, 82%), 30% of students found this feedback to be effective compared to 18% at Auburn University and 25% at The University of Sydney.

#### Interactions

Students were comfortable participating in the live online sessions during ERI (Questions: Table 2-row L-Q1, Results: Table 10). Students at The University of Sydney found asking questions to be somewhat or much better online compared to in-person (46% compared to 22%), while at Auburn University this was reversed and only 22% found it somewhat or much better while 36% found it somewhat or much worse (Questions: Table 2-row L-Q2, Results: Table 10). There was no difference due to gender in comfort in participating in live online sessions at The University of Sydney (p = 0.97), while there was a gender difference with males being more comfortable at Stockholm University (p_sto_ = 0.024).

**Table 10:** Responses of students to a question “My comfort level in asking questions in this online course compared to my previous in-person college courses was” on a Likert scale from much better to much worse during the COVID-19 pandemic at Auburn University, United States, and The University of Sydney, Australia. Data is percentage and there was a total number of 1,081 responses.

Likert Scale descriptions	Auburn (n = 960)	Sydney (n = 121)
Much Better	9%	17%
Somewhat Better	13%	29%
About the Same	41%	32%
Somewhat Worse	18%	8%
Much Worse	18%	14%

The least common interaction strategy used by students during live sessions was to ask questions by raising hands, and the most common strategy was to ask questions or make comments through the chat box function (Questions: Table 2-row L-Q3, Results: Table 11). For those students who did raise their hands to ask questions, we observed a gender difference, with males four times more likely to raise their hands to ask questions compared to females (p = 0.04). The most common interaction strategy used by students to interact with instructors was through email followed by the discussion board (Questions: Table 2-row L-Q4, Results: Table 12A and 12B) and many students did not interact with their instructors during live classes or outside class (Table 12A and 12B). There was no significant gender difference in the overall number of interactions either in live classes or outside class, although as indicated earlier, males were four times more likely to ask questions during live sessions compared to females. A large percentage of students spent less than 10% of their time with other students outside of class, 53% and 65% respectively at Auburn University and The University of Sydney. An even greater number of students, 76% at Stockholm University, did not spend time with other students outside of class (Questions: Table 2-row L-Q5). There were no differences due to gender (p_syd_ = 0.12, p_sto_ = 0.063).

**Table 11:** Most and least common interaction in live online sessions during the COVID-19 pandemic at Auburn University, United States, Stockholm University and The University of Sydney, Australia. Data is percentage and there was a total number of 1,856 responses. * Indicates a significant effect of gender, where males were four times more likely to ask questions raising hands than females.

Ranking	Auburn (n = 960)	Sydney (n = 121)
Did not ask questions	51%	25%
Asked questions		
• Raising hands*	14%	10%
• Chat	55%	55%
• Breakout rooms	22%	46%

**Table 12:** Mode of interactions between instructors and students in (a) live online classes and (b) outside classes during the COVID-19 pandemic at Auburn University, United States, and The University of Sydney, Australia. Data is percentage and there was a total number of 1,081 responses.

a.		
Mode during live classes	Auburn (n = 960)	Sydney (n = 121)
Did not interact	35%	35%
Office hours	13%	3%
Zoom outside office hours	8%	3%
Discussion Board	7%	20%
Emails	37%	31%
b.		
Mode during outside classes	Auburn (n = 960)	Sydney (n = 121)
Did not interact	57%	38%
Office hours	10%	7%
Zoom outside office hours	7%	7%
Discussion board & emails	15%	35%

Overall, students reported that the ease of interactions between instructors and students and student to student interactions diminished during ERI. Students at Auburn University (43% and 53%) reported it was more difficult to interact with instructors and students, while this was easier at Stockholm University (23% and 29% of students) (Questions: Table 2-row L-Q6, 7 and 8, Results: Table 13). Overall, while not all students reported that interactions with peers became more difficult because of ERI, our results indicated that these interactions were compromised.

**Table 13:** Ease of interactions between instructors and students and students to students during the COVID-19 pandemic at Auburn University, United States, and Stockholm University, Sweden. Data is percentage and there was a total number of 1,735 responses.

Ease of interactions	Stockholm (n = 775)	Auburn (n = 960)
Between instructors	23% found it harder 55% found it similar	43% found it harder 41% found it similar
Between students	29% found it harder 53% found it similar	53% found it harder 34% found it similar

## Discussion

### Responses of instructors

Overall, this study found that instructors rapidly shifted to ERI across culturally and geographically separated institutions. Similar to before the COVID-19 pandemic, instructors primarily used lectures, graded assignments and mid and final semester exams as the main instructional strategies. While instructors shifted lectures to online during ERI, they were still passive rather than active lectures with little interactivity. Instructors still expected students to watch pre-recorded lectures if they needed to clarify understanding, but few, if any, checked on students’ understanding from these lectures. Problematically, at Auburn University lectures were unrecorded so students could not return to them later. In contrast, lectures were routinely recorded pre and during the COVID-19 pandemic at The University of Sydney and students could return to them later.

In the unique context of ERI, the lack of recorded lectures and no follow up on student understanding may have been problematic for the mental health and well-being and performance of students. Other studies have reported that during ERI students had difficulty maintaining attention, were unable to use resources effectively and consequently had difficulty constructing meaning from the course materials, and ultimately experienced mental health and well-being issues such as increased anxiety [34].

While it is reasonable to expect that instructors would make changes to the ways in which they assessed students or the percentage weighting that high-stakes assessments contributed to students’ final course grades, to reduce student anxiety, the results of this study showed that this was not the case. High-stakes assessments and final and mid semester exams remained a significant weighting of the final course grades at Auburn University and at The University of Sydney. At these institutions either Proctor U or other online invigilation tools were used to secure academic integrity. At Stockholm University, where exams are not as common, assessments also remained unchanged.

Previous studies pre pandemic have found student experiences with Proctor U as unsatisfactory because of technical difficulties and personal issues with proctors [35]. Milone et al. [35] found just over half of student indicating that the use of Proctor U would impact their course choice. Studies done on the impact of Proctor U during the COVID-19 pandemic found that students experienced stress and anxiety over Proctor U exams and were concerned about being monitored via a webcam [36]. Moreover, during the pandemic many students were “Zooming” in from their bedrooms which were sometimes a shared space which made learning and assessment more problematic [37, 38]. Academics were often also “Zooming” from a shared space [38, 39] with partners and with responsibilities for children who were also learning at home [3, 4].

According to pre-pandemic research [40], online assessments are most effective when they provide ample feedback to students. While this study did not ask instructors their views specifically on feedback, the data suggest an emphasis on summative assessments with little feedback. It is well documented, however, that even when students receive feedback on assessments, they find it unhelpful or do not reflect on it appropriately. Many studies have reported that providing effective feedback is one of the most challenging areas for the higher education sector [41–44]. Given that the COVID-19 pandemic did not change instructional strategies or assessment it is unlikely that assessment and feedback has changed now that learning has returned to campus.

Unsurprisingly, interactions between instructors and students were infrequent during the COVID-19 pandemic [45]. In fact, the most common response to a question about the types of interaction students engaged in was ‘no interaction’. Most instructors did not monitor group discussions or poll students to regularly gauge student engagement. The lack of interaction found in our study aligns with other studies that also found reduced engagement levels among students during ERI [45–48]. The most common interactions between instructors and students at Stockholm University and The University of Sydney were through feedback provided on their assignments or exams and at Auburn University, the most common interaction occurred during the instructor’s office hours.

The finding that interactions between instructors and students were also concerningly low prior to ERI was surprising. Although most instructors were proactive in setting up virtual office hours to engage with students more regularly during ERI than during in-person semesters, this study found interactions were less than 10%. Interactions during ERI were virtually no different when compared to the previous in-person on campus semesters. Perhaps this was because of a perception that one is not ‘interacting’ with students during virtual office hours over a computer (often with cameras turned off). Other studies have also identified dissatisfaction with interactions in online learning including poor communication with instructors as well as an inability to collaborate effectively with peers [49].

This study also found that a third of students did not interact with instructors during synchronous live online classes. Instead, students interacted with instructors more frequently through email. This is aligned with other studies, which also found a lack of interactions with students, and reports of student social isolation being one of the main challenges of ERI and the COVID-19 pandemic [6]. In a post COVID-19 campus, there is a clear need to implement activities in-person and in online virtual spaces that facilitate interactions between students and instructors. It is evident, therefore, that student engagement and interactions between academics and students should be a critical area of future research now that students are back on campus.

This study also found that while instructors at all universities agreed or strongly agreed that they can comfortably teach online, they did not prefer to teach online. Perhaps this was because the rapid shift from face-to-face teaching to ERI did not allow instructors or faculty to prepare adequately [6]. It may be that these negative attitudes about online learning were because students and instructors surveyed in this study were in science courses which required ‘hands-on’ practical experiences, such as fieldwork [50] and laboratories that could not be effectively delivered in ERI [51, 52]. As stated in the introduction, previous studies have found that central to successful online learning is the design of learning environments with three-way interactions, where students interact with content, each other (peers) and instructors [2, 6, 16]. There is greater difficulty with peer-to-peer interactions in synchronous online learning, especially with cameras off as well as difficulty in engaging with noninteractive asynchronous lecture.

Overall, instructors were eager to safely return to face-to-face teaching. Although some ERI instructional components can be viewed as effective [50, 53] asynchronous instruction that involves little to no interaction did not engage instructors or students sufficiently [54]. This is not surprising, as the pivot to ERI required instructors to teach online regardless of whether they had previous experience of teaching online. Instructors were also expected to learn how to teach online within a matter of days, an unrealistic timeframe [55].

In summary, this study shows that most instructors responded to the COVID-19 pandemic and ERI by transferring in-class teaching into the online format, which not only led to suboptimal instructional designs but also resulted in instructor and student dissatisfaction.

### Responses of students

Across all three institutions, students reported that ERI reduced education quality. In response to ERI, students in all institutions reported that they created and followed a daily study plan, although there were some differences. For example, more students at Stockholm University reported that they had and followed a daily plan compared to students at Auburn University and at The University of Sydney. Fewer students at Stockholm University reported that they were distracted by conditions such as the physical environment or the internet, that hindered online learning of students at the other two universities. These findings are consistent with other studies that explored the experiences of students around the world [54, 56], with reports of positive and negative learning experiences during ERI [47, 53, 57, 58]. Positive aspects of ERI for students included the comfortable home environment, efficient time use, interactions with instructors [48, 56], flexibility, convenience [47] and the freedom to structure their learning [45, 59]. Negative aspects included anxiety and a feeling their learning was compromised, even when the transition to on line learning was smooth [58].

In this study, students ranked passive watching of lectures and recordings, completing assignments, and watching live lectures, all of which are a form of surface learning, as the most effective tasks for their learning. At Auburn University and The University of Sydney students ranked watching recorded videos, completing assignments, and watching/participation in live lectures highest, while students at Stockholm University ranked watching recorded videos, reading, and watching live lectures as the most effective. Some of these small differences could have been because students at Stockholm University had more regular homework assessments with the five-week course structure and less emphasis on assignments. When lectures and other instructional strategies, however, were live and synchronous, a high percentage of students had their cameras off and appeared to be disengaged. Similar findings were reported in other studies [60, 61] and may have contributed to learning experiences [56–58, 62]. Ewing and Cooper [57] found “students found online learning to be less personalised. While the pandemic has expedited emergency technology adoption in schools, this is not equivalent to the purposeful integration of technology” (p. 41).

Negative aspects of ERI included the more effortful, active learning tasks such as study groups and instructors’ virtual office hours, which were ranked as least effective for student learning. These negative responses to active learning strategies are a concern given that previous studies have found that active study time and engaged behaviors improve understanding and exam performance [63]. In our study we did not measure and are thus unable to determine the extent that students who rated active behaviors as ineffective were actually participating in those activities. During live class sessions, a large percentage of students in all institutions appeared to be disengaged with cameras off and with no participation in discussions. This study also did not collect evidence that could be used to determine student engagement in and performance. This research was limited in that we were unable to collect data on student performance. This means we cannot speculate about whether the lack of active learning strategies may have impacted student performance.

Students reported the same or increased levels of comfort in asking questions in the online environment compared to in-person classes, highlighting the importance of the chat window feature, where students can type in a question during a lecture. These findings align with Shim and Lee [48], who also found that students value the chat function. While students have the option of raising their hand to notify the instructor they have a verbal question, this option was used less frequently compared to the chat window. Monitoring the chat window while teaching can, however, be challenging for instructors. When the chat room was monitored, researchers have found that they are more likely to be dominated by males compared to females [69]. This is in agreement with a significant number of previous research studies showing males more likely than females to voluntarily participate and ask questions in classrooms [64–70]. For example, Nichols et al. [68] found that during the pandemic males participated more than females, both verbally and using the chat window, during online lessons. In this study, males were found to be four times more likely than females to raise their hands and ask questions. There is, however, little research about gendered participation which simultaneously compares both the face to face and online environments, but the few studies done to date have found gendered patterns. Caspi et al. [70] found males spoke more in in-person classrooms whereas females over-proportionally posted messages in online environments.

Nichols et al. [68] also found that chat comments posted by male students were acknowledged by peers more than those posted by female students. They recommended that instructors use a greater variety of Zoom features available such as breakout rooms and polls to lower barriers for participation for all students. Similarly, Caspi et al. [70] recommended that to lower barriers for participation for all students, instructors use a greater variety of Zoom features available such as breakout rooms and polls. These findings suggest that further research is required to investigate how to foster inclusive participation.

Overall, interactions between students and instructors were low in all institutions during ERI. Concerningly, these results suggest that the frequency of instructor-student interaction outside class did not, however, change drastically because of ERI as interactions were also not frequent prior to the COVID-19 pandemic. This low frequency of interactions outside class were particularly problematic during ERI, as there were also few interactions with students during live synchronous sessions or no live session altogether.

Accessibility of instructors or peers differed depending on the university. Most students at Stockholm University benefitted from existing university-sponsored software to create an online community network and reported similar levels of interactions with instructors and peers during ERI. Students at Auburn University and The University of Sydney did not have access to similar online software and found it harder to interact with instructors and peers during ERI [71].

Not surprisingly, students at both Auburn University and The University of Sydney found the quality of interactions diminished during ERI. An important conclusion to be made is that online software which build community networks are highly beneficial for online learning.

This study did not find evidence that technology issues such as incompatible devices, inconsistent supply of electricity, unstable internet network and costly data were barriers to learning during ERI. Instead, it supports other research that students experienced distractions from social media and suboptimal home environments [72, 73].

### Global perspectives

The majority of studies on the impact of COVID-19 pandemic and the move to ERI on learning in higher education analyzed individual courses or multiple courses in the same institution [10, 36, 48, 60]. This is one of the first studies to analyze multiple courses in science at multiple institutions. Our results are thus more generalized and more broadly applicable than studies based on a single course at a single university. Online learning environments in these disciplines have the distinct disadvantage of not being able to facilitate/support field work or practical investigations in a range of disciplines [51, 52].

Given the rapid shift to ERI, the COVID-19 pandemic made it logistically difficult to develop multi-institutional, collaborative research projects that address similar questions across multiple cultural contexts. However, by remaining siloed, the extent to which data trends and effective online strategies are generalizable across multiple contexts remain unclear. In our study, we considered the potentially distinct cultures, norms, and values within each institution that may drive observed results across three universities on three continents: Stockholm University in Stockholm, Sweden, Auburn University in Alabama, United States, and The University of Sydney, Australia. Although there are differences between universities based on geographic region, campus culture, student body composition, history, and other factors we found commonalities, especially in relation to learning resources and interactions between instructors and students during ERI.

## Conclusion

Never before has higher education, globally, been faced with the need to move to online learning in a matter of days. This study has found that although higher education institutions vary in terms of their cultural constructs and geographic global locations [28–31, 74, 75] all experienced similar challenges during ERI. Overall, ERI was successful in sustaining learning which otherwise would not have been possible in the face of the COVID-19 pandemic. Still, the results of this study paint a concerning picture of ERI as a disengaging non-interactive learning environment. One which was deprived of instructor and student interactions, reduced collaborative opportunities, an absence of formative assessments to gauge students’ learning, and effective and timely feedback from instructors.

Although there were some valuable resources developed for learning science [60–76], these are not replacements for practical laboratories which are known to develop cognitive and psychomotor skills [77, 78] or for other disciplines such as Art where technique development is important [79]. Prior to the COVID-19 pandemic the lack of interactive pedagogies and strategies [65, 66] were also a concern for instructors and institutions. The impact of the COVID-19 pandemic and ERI has been to focus on the significance of interactions and the need to improve education quality, implement effective pedagogies and a greater need to focus on the student experience and inclusivity [80].

A growing body of literature underscores the lingering and long-lasting negative impact of the global COVID-19 pandemic and ERI on higher education. This study offers stakeholders such as educational designers, learning and teaching experts and higher education management a comparative picture of instructors’ and students’ experiences in three institutions which used vastly different ERI policies, across three continents. Importantly, it reinforces the need to increase student engagement and interactions with peers and instructors on campus and to prepare for future disruptions to learning, in higher education, which are no doubt yet to come.

## Supporting information

S1 Table Description of Universities in the study. (DOCX)
