# Supplementary material for: Global perspectives of the impact of the COVID-19 pandemic on learning science in higher education
Source: PLoS One. 2023 Dec 7;18(12):e0294821. doi: 10.1371/journal.pone.0294821 (PMC10703257; doi:10.1371/journal.pone.0294821)
Supplement: S1 Table — (DOCX) [file pone.0294821.s001.docx]

Supporting Information

S1 Table Description of Universities in the study

**Sweden- Stockholm University**

*Summary description*

Stockholm University is one of Sweden’s largest universities, with 27,000 full-time-equivalent students and 5,000 members of staff distributed over more than 50 academic departments and central administration (HR, IT-support, library, international office etc.). The university is divided into four schools/faculties: humanities, social sciences, law andnatural sciences. Swedish universities have a two-semester system, where the academic term in Sweden runs September to mid-January and end of January to June. Courses are generally divided into blocks of approximately 5 weeks, but there are also both shorter and longer courses. Most students take one course at a time and one term of studying adds up to 30 ECTS (20 weeks), although it is possible to be registered for an additional 15 ECTS. The courses usually run full time, expecting 30-40 hours work per week, or half time. Most courses are part of undergraduate or graduate (PhD and masters) programs, but there are also ‘freestanding’ courses that you can study outside of an educational program and constitute your own study profile.

*Academic careers*

When applying for a full-time position as lecturer or professor applicants must attach a scientific and a teaching portfolio which are assessed by external experts of the discipline. However, the employment regulations of the university do not specifically state the criteria for how to evaluate the qualities of the teaching portfolio whereas the qualities of the scientific portfolio is rather clear. As a consequence, one of the faculties (School of Law)has stated their own criteria for assessment and also use specifically appointed experts to assess the teaching portfolio, something that is partly followed by the School of Natural Sciences. The Swedish universities have an agreement through the association of Swedish Higher Education Institutions, SUHF, that for full-time teaching positions formal higher educational training corresponding to ten weeks of full-time studies is normallyrequired.

*Teaching and courses*

In this section we describe what happened at Stockholm University when the COVID-19 pandemic hit Sweden.

*The case of Stockholm University*

During the week of the winter break in the Stockholm area, and when many Stockholm based families go skiing in Italy, Switzerland and Austria, alarms regarding the outbreak of COVID-19 spread. Back at work in early March 2020 departments started to discuss strategies.Mid-March, recommendations were sent out to prepare for online teaching. On March 17, the prime minister announced that all high school and all higher education teaching would go distance based, effective from March 18. The state epidemiologist held daily press briefings at this time as well with updates that might need action.Late April, the Public Health Agency made it possible to give exemptions to online teaching and examinations, for practical session. Decisions on which exemptions to grant were taken by the science areas upon application from the department. Early June, it was possible to make exemptions also for other physical teaching and examination than practical examinations.*Emergency groups*

At Stockholm University, a few emergency COVID-19 groups were constructed. One group focused on administration, and general rules and recommendations. Another focused more on teaching and education and recommendations and rules about these. The aims of the groups were to be able to act swiftly and take the necessary decisions (on the right levels). The central communications section was also part of the group to formulate both internal and external communication and follow media and other universities' decisions. These groups met almost daily in the beginning.

A collaborative group consisting of leadership representatives, teaching and learning experts from the Centre for Advancement of University Teaching, and representatives from the IT-department was constructed with the aim to safeguard issues of importance for the quality of teaching and learning. The group made sure a play service to enable video material in a non-commercial environment for teaching was established. Such a service did not exist before although it had been asked for by some teachers doing distance or blended education for quite some time.

*Strategy*

The university's strategy was to follow the decentralized structure of the university and only general rules were decided by the Rector. Most other concrete decisions about ongoing and planned courses were done at the department level. The science areas provided recommendations and guidelines for the departments. Video recorded presidential ‘talks’ to all members of staff were organized as a way to keep in contact and inform about news.*Resources for teachers*

A website called ‘teaching online’ was constructed with regular updates on resources, tips and information. **The** Centre for the Advancement of University Teaching (CeUL) started sending emails to pedagogical contact persons in all departments twice a week, to be forwarded to all teaching staff, with links to the ‘teaching online’ website. The website was linked to by the communication department of Stockholm University, and for the first time the communication department, the IT department and CeUL started to collaborate more closely regarding information provided to university staff. Stockholm University’s planned biannual teaching and learning conference was cancelled at the last minute and replaced by a half-day workshop focusing on teaching at a distance. The content focused mainly on technical aspects of how to use the video conferencing system Zoom in teaching, but also a few workshops focusing on pedagogical issues of student learning in a distance educational environment. Drop-in sessions to learn Zoom via Zoom were organized for teachers several times a week y CeUL. This was a new activity and generated quite a few participants during March-April.However, as teachers’ competence and confidence grew, the needs changed into focusing more on pedagogical questions rather than technical. Hence, half day workshops with parallel sessions were organized at the beginning of May focusing on distanced based teaching, online teaching principles and assessments online etc.

*Change of exams*

It became clear rather quickly that the exams needed to change into home-exams, home assignments etc. to a large degree. Still quite a few courses used sit-ins in front of the web camera, or just did them without any kind of screening. Some exams were cancelled, but since the sitting of exams and getting credits registered in the system is crucial for students in Sweden being able to receive their student loans, and for the departments to get paid for their teaching, this was seen as a last way out. However, there was a fear of students cheating at exams, something that also was confirmed as there was a 50% increase of disciplinary cases during 2020 than for 2019. The cases were dominated by plagiarism and prohibited collaborations in home exams.

*Infrastructure adaptions*

In a few days the number of possible video meetings (Zoom) had to increase its limit by the

IT-department from 4,000 to 120,000 in the Nordic countries. Before this limit had been changed, numerous teachers and students experienced being locked out of meetings, workshops, and lectures that were moved online. A lot of teachers transferred their normal

lectures to Zoom, some let students deal with the reading lists instead. Only a few recorded

parts of, or full lectures and put them via the play service organized in April. However, the

increase in the number of open lectures has been steep.

Athena, the learning management system (LMS) used by Stockholm University (and by many

schools in Scandinavia) had to increase its capacity of online users in a short space of time.

For courses where the LMS was already in use and the design was more or less ‘blended’ the

changes to the courses were not huge. Courses that were lecture based however, experienced a big change, as did the assessments that had planned for sit-in exams.

**USA- Auburn University**

***Summary***

Auburn University is a comprehensive land-grant institution located in Auburn, Alabama, United States. With more than 24,600 undergraduate students and approximately 1,330 faculty members, Auburn is among the largest universities in the Southeast United States. It is one of the state's two public flagship universities, along with University of Alabama. Auburn includes the College of Sciences and Mathematics (COSAM), comprised of five departments: Biological Sciences, Chemistry and Biochemistry, Geosciences, Mathematics and Statistics, and Physics. COSAM comprises approximately 200 faculty members, more than 2,700 undergraduate students, and nearly 400 graduate students. COSAM includes professional degree programs in nursing, pharmacy, and veterinary medicine.

*Auburn, Alabama: a college town.* Though Auburn’s student body is mostly composed of students from the state of Alabama (37% according to Fall 2017 summary statistics), approximately 57% of its students are from states outside of Alabama. Approximately 6% of the student body are international students. Auburn is considered a ‘college town’ because the vast majority of students moved to Auburn to attend college and the community is dominated by its university population. In fact, only 5% of the total student body are from the immediate Auburn area or close surrounds (i.e., Lee County, Alabama).

*Teaching staff*

Teaching staff at Auburn University consist of tenure-track faculty (i.e., assistant, associate, or full professors) and lecturers (non-tenure track instructors for whom teaching is their main task). Assistant professors generally serve five complete years on full-time appointment before they may be nominated for tenure and promotion to associate professor. Tenure and promotion to associate professor rank is on the basis of excellence in teaching, research or other creative work, outreach activities, and service to the university. Standards for promotion and tenure are based on the weights of each performance area as described in the offer letter. Updates on progress are communicated in annual evaluations. Notably, while excellence in research follows a set of competitive and objective criteria (Auburn is classified as "R1: Doctoral Universities – Very High Research Activity"), evaluation of teaching is largely conducted through student evaluations and unstructured faculty-peer reviews. Lecturers have the opportunity to move up in rank as senior lecturers through a similar process of relatively subjective evaluation of teaching.

*Teaching resources.* Auburn University houses the Biggio Center which has a mission to engage and support the academic community in scholarly, evidence-based teaching. Support units include Educational Development, Instructional Technology Testing Services, and Learning Experience Design*.* Prior to the pandemic, the Biggio Center supported faculty teaching through workshops, consulting, and use of emerging instructional technologies such as personal response systems and active learning classrooms on campus. These engaged, active student learning (EASL) classrooms are scattered throughout the campus, and the most recent and currently largest set of new classroom spaces includes 29 active learning classrooms seating between 15 and 64 students; and two active learning large lecture halls seating 166 students each. Instructors relied on the Biggio Center staff after the COVID-transition to remote instruction because many had never engaged in online teaching, testing, or used online proctoring software.

*Timeline of COVID as it impacted Auburn University*

In this section we describe the series of events surrounding Auburn University’s transition to remote instruction during the spring 2020 semester. Spring semester lasted from January 8 to April 24, 2020. The text is written from the perspective of one faculty member who was teaching a large introductory biology class. The text is based on their experience and informed by emails and announcements from administrative decision-makers.

*The sudden switch to online teaching in March 2020*

Auburn students, faculty, and staff were informed that a university task force had assembled to monitor the COVID-19 virus and its potential impacts on the University in late February 2020. At the time, there were no known cases in the state of Alabama. However, by March 2, Auburn University recalled students studying abroad, suspended all international travel, and braced for spring break, a school holiday that took place in mid-March. It was during this break, on March 12, that University officials informed the community that starting March 16, Auburn University will transition from on-campus instruction to remote delivery in response to concerns about the spread of the COVID-19 virus. Additionally, University officials asked that students not return to campus after the spring break holiday, which concluded March 16. At the time, reports of the decision indicated further information was to be made available by April 10 that will determine if students returned to campus for the remaining weeks of the spring semester. By that April deadline, the University President and Provost announced that all on-campus events were cancelled through the end of the spring semester. Thus, students continued with remote instruction and did not return to campus at all in spring 2020.

*Teaching strategy*

University officials instructed Auburn faculty to communicate with students on steps to continue their academic coursework remotely. Instructors relied on CANVAS, a learning management system used by Auburn University. Students signed into CANVAS to obtain course information, lecture content/documents, submit assignments, post on discussion boards, take exams, watch zoom lectures, and more. Auburn also used Zoom software, a cloud platform for sharing video and content that runs across mobile devices, desktops, telephones, and other systems. The university's strategy allowed individual departments to decide which mode of instruction best suited their disciplines, and faculty were encouraged to use instruction that made most sense for their courses. Auburn - perhaps more than other institutions - had to offer maximum flexibility because the vast majority of students did not live in the Auburn area; according to 2017 admissions information, prior to enrolling at Auburn University, approximately 95% of students lived outside of the Auburn area and surrounds (i.e., Lee County) and 63% of students lived outside of the state of Alabama. To accommodate students, many instructors elected to use remote *asynchronous instruction*, an approach characterized by teaching and learning that does not occur at the same time. For example, after the transition to remote instruction, one student contacted one of the authors (Ballen) who taught biology at 8:00 am on Tuesdays/Thursdays, informing her that she was located at her parents’ house in California for the remaining duration of the spring semester. She expressed concern because *synchronous* virtual instruction – in which teaching and learning occurs at the same time - would require she attend virtual class sessions starting at 6:00 am her local time. While imperfect and not optimal for learning, most instructors converged on the idea that asynchronous lectures were the best solution for a bad situation. It allowed for equitable access to instruction and lectures for all students. Some instructors pre-recorded lectures, while others delivered live lectures at their normal time, but uploaded them for students to watch when they were able.

*Change of exams*

Exams posed another challenge for instructors in spring 2020, particularly for those who taught large lecture courses. Instructors largely relied on remote proctoring technology such as Honorlock or ProctorU, both supported by Auburn University and integrated into our learning management system (i.e., CANVAS). While previous research shows remote proctoring decreases cheating and other forms of academic dishonesty during exams, electronic performance monitoring literature suggests computer-based surveillance increases anxiety . Despite potential shortcomings, classes relied on remote proctoring because most classes within COSAM used closed-note and multiple-choices exams, and because face-to-face proctoring was not an option.

**Australia- The University of Sydney**

***Summary***

The University of Sydney is Australia’s oldest university. Founded in 1850, it is a public, research-intensive university located in Sydney with campuses in the city suburbs of Camperdown and Darlington and across western Sydney including Westmead and Camden. It has 73,000 students, 8,100 academic and professional staff and 380,000 alumni in more than 170 countries. It is consistently ranked as one of the top 50 universities in the world and is known as one of eight ‘sandstone’ universities in Australia, named after many of the original buildings which are made from sandstone. The University of Sydney has a focus on research, known as a research-intensive, Group of Eight, universities in Australia. The focus of education curricula is on building expertise in the disciplines. It has an outstanding reputation of past alumni including prime ministers and is ranked first in Australia for graduate employment. In recent changes to the curriculum, the bachelor’s degrees have become more contemporary, interdisciplinary, and connected to the world of work. The University of Sydney has eight faculties and schools, two of the largest are the Faculty of Medicine and Health and the Faculty of Science. The Faculty of Science has a diverse range of educational programs including professional degrees of veterinary medicine in the Sydney School of Veterinary Science, through to concentrations in Life Sciences, Geosciences and Mathematical and Physical sciences.

*Teaching Staff*

Teaching staff at The University of Sydney consist of tenure track academic staff who are either in research, teaching and research, or education focused positions. Research focused staff are either tenure-track faculty (i.e. associate or full professors) or lecturers who may either be on tenure track or on external research fellowships. Most academics in Australian universities are employed in traditional teaching and research roles. Academic roles which are both teaching and research, are generally defined on the proportion of activities, spent on research, teaching and community or governance, the percentage of contributions into these activities are 40% teaching, 40% research and 20% governance. Often this role is referred to as the 40:40:20 model. Research intensive universities such as The University of Sydney, or the Group of Eight (Go8) universities have the majority of academics either in full time research or who mix research and teaching. The academic role, which is growing at the fastest rate is that of the education focused academic. Over the past decade, education/teaching focused roles have accounted for around 50% of growth in academic roles, whereas traditional teaching and research roles accounted for only 15%, and research focused roles accounted for 35% of growth in this period. Academics in education focused positions are evaluated using a broad range of metrics, such as the [Australian University Teaching and Criteria and Standards Framework](http://uniteachingcriteria.edu.au/) which was designed to provide academics with a practical guide describing quality teaching and how it can be evidenced, and the [Queensland University of Technology (QUT) evaluative framework ‘Reframe’](https://eprints.qut.edu.au/70534/1/reframe-approach-at-a-glance.pdf) which provides academics with a reflective tool to design, deliver and evaluate learning experiences. The metric perhaps most used in Australia is the United Kingdom [Professional Standards Framework Higher Education](https://www.heacademy.ac.uk/ukpsf) from the Higher Education Academy (HEA UK, now Advance HE). This is an internationally recognised framework which aims to raise the profile of learning and teaching in higher education. It represents standards against which academics can describe their professional practice, so as to pursue fellowships at various levels.

*Teaching Resources*

The University of Sydney has an Education portfolio with an emphasis on approaches to better prepare students for the increasingly competitive, fluid and challenging work environments of the future. Commitments include interdisciplinary opportunities and leadership opportunities for all students, as well as experiential and authentic learning. There are nodes of educational innovation and learning resources in libraries specific to disciplines and professions across campuses of the university. A university wide curriculum which commenced in 2018, created small ‘bite sized’ online learning units, called the Open Learning Environment. Staff across the university involved in the design and creation of these units were perhaps those most prepared for the COVID-19 pandemic.

*Timeline of COVID as it impacted the University of Sydney*

In this section we describe the series of events surrounding The University of Sydney’s transition to remote instruction in 2020. For most of the university, semesters are 18 weeks in length, generally with a commencement in February and August of a calendar year. In 2020, semester 1 commenced with a welcome week beginning on 24 February, classes started on the 1 March and finished on 26 of June. On 29 January, the Australian government announced a travel ban for international arrivals, including students from China, Iran, Italy and South Korea. Initially, the travel ban was for specific countries and at first there was a sense it would be short; it was extended in two-week intervals, providing hope that borders may reopen. However, as international community transmission of COVID-19 accelerated, by 20 March the travel ban had been extended to include all international travellers and commencing students..The travel ban meant that returning students who were currently out of Australia were unable to re-enter Australia. The way the travel ban was initially implemented i.e., put in place for 2 weeks, with extensions of 2 weeks, made planning for learning and teaching very difficult. The university explored several options, which included extending the time for students to arrive in Australia until 30 March. Classes commenced on 24 March with face-to-face teaching for domestic students and online teaching for international students who could not be in Australia. A week later, however, it was clear that it was not only international students whose learning would be impacted by COVID-19. COVID-19 was spreading through the Australian community. The university decided, on 13 March, for all teaching and learning to be moved incrementally online, with a fully online delivery to be in place by 23 March. The very next day, on 14 March, the first COVID-19 case had been identified on campus and by the 15 March, all learning and teaching was delivered online for students in the Faculty of Science. Students continued with remote online teaching until August 2020. Unlike American and European universities, most domestic students in Australia attend their local university and live with their parents. In the Faculty of Science, 88% of students are domestic and around 11-12% are international students.

*Teaching strategy*

Academics were primarily responsible for deciding how to best deliver learning and teaching remotely to students using CANVAS, the relatively new learning management system introduced to the University of Sydney in 2018. Teaching and learning took place through CANVAS and the Zoom software application, using resources which had been created either in synchronous or asynchronous mode. The faculty of Science created a COVID-19 response group facilitated by an education lead, who was responsible for communicating rapid changes to assessments and exams to instructors and educational leads within the nine schools within the faculty. Importantly for this study, because the academic year commenced in the last week of February and online remote teaching and learning commenced on 15 March, students who could make it to campus, only had three weeks to get to know each other and international students had no interactions with other students in their courses.

*Change of exams*

Exams were a challenge to organize at the end of semester 1 2020. At first there was denial that face-to-face exams were not possible as scheduled in July 2020. There was a belief that the emergency would be over by this time. When the World Health Organisation declared, on 11 March, COVID-19 to be a pandemic there was a realization that online exam technology would be required. Proctor U was selected as the vendor because the service could be integrated into our learning management system. Students in Australia were very resistant to such technology intruding into their private space. In response to these concerns, several faculties within the university implemented open book, problem-based assessment, rather than using the Proctor U platform. This was challenging, because instructors were not used to writing problem-based questions nor creating exams in CANVAS. In response, the Faculty of Science implemented a Quality Assurance peer review process, which ensured that the exam questions reached the required standard of quality and that exams were technically possible to complete using CANVAS. In total, over 2020, the Faculty of Science managed 250,000 sittings of open-book and problem-based exams, with minimal academic integrity breaches detected. The Faculty also had a selection of proctored exams, primarily for students who were in their final semester or with professional accreditation requirements.
